# Very low sensitivity of wet mount microscopy compared to PCR against culture in the diagnosis of vaginal trichomoniasis in Uganda: a cross sectional study

**DOI:** 10.1186/s13104-017-2581-1

**Published:** 2017-07-06

**Authors:** Sheila Nabweyambo, Othman Kakaire, Stefanie Sowinski, Alfred Okeng, Henry Ojiambo, Joshua Kimeze, Irene Najjingo, Freddie Bwanga

**Affiliations:** 1 Department of Medical Microbiology, Makerere University College of Health Sciences, P.O. Box 7072, Kampala, Uganda; 2 Department of Obstetrics/Gynaecology, Makerere University College of Health Sciences, P.O. Box 7072, Kampala, Uganda; 3 Infectious Diseases Institute, Makerere University College of Health Sciences, P.O. Box 22418, Kampala, Uganda; 4 MBN Clinical Laboratories, P.O. Box 35135, Kampala, Uganda; 5 Sexually Transmitted Diseases Clinic Mulago Hospital Kampala, Kampala, Uganda

**Keywords:** *Trichomonas vaginalis*, Sexually transmitted infections, Trichomoniasis, Wet mount microscopy, Polymerase chain reaction

## Abstract

**Background:**

*Trichomonas vaginalis* (TV) causes the Trichomoniasis Syndrome composed of vaginitis in women, urethritis in men and tube infection in both sexes. This infection is strongly associated with premature rupture of membranes, preterm delivery, low birth weight, promoting HIV sexual transmission and infertility. Prevention of these complications requires accurate early detection and effective treatment of infected individuals. In the resource limited settings, the wet mount microscopy (WMM) is often the only available test for laboratory detection of TV, but its accuracy and that of polymerase chain reaction (PCR) tools in Uganda remain poorly studied. The aim of this cross-sectional study was to compare the diagnostic accuracy of the WMM and PCR against culture as reference standard for the direct diagnosis of TV among symptomatic women. Three high vaginal swabs were collected from each of one hundred fifty women presenting with symptoms suggestive of active vaginal trichomoniasis at the sexually transmitted diseases clinic of Mulago National Referral Hospital Kampala, Uganda. The swabs were tested for TV with WMM, in-house PCR and TV culture. Results were analysed using excel 2007, SPSS v16, and Meta-disc software to determine the diagnostic accuracy of the tests.

**Results:**

The sensitivity, specificity and kappa agreement of the WMM was 25% (95% CI 5.5–57.2%), 100% (95% CI 97–100) and 0.38, respectively. Corresponding values for the PCR were 91.7% (95% CI 61.5–99.8), 99.3% (95% CI 96–100) and 0.91, respectively.

**Conclusion:**

Among the TV symptomatic women, the sensitivity of the WMM was very low, with two-thirds of the patients missing a diagnosis while the in-house PCR was highly sensitive and specific. Feasibility studies aimed at incorporating PCR tools in algorithms for diagnosis of TV infection in resource-limited settings are recommended.

**Electronic supplementary material:**

The online version of this article (doi:10.1186/s13104-017-2581-1) contains supplementary material, which is available to authorized users.

## Background

*Trichomonas vaginalis* (TV) is an extracellular, facultative anaerobic infection of the human urogenital tract. Infection is associated with premature rupture of membranes, preterm delivery, low birth weight, HIV sexual transmission [1], and pelvic inflammatory disease [2–4]. The WHO estimates that at least 174 million cases of infection are acquired annually worldwide, majority in regions of low income settings [5, 6]. The prevalence of TV infection differs in different subpopulation groups, e.g. in women, it is estimated to be ten times higher than in males [7, 8]. In the USA, 13% of black women are affected compared with 1.8% of non-Hispanic white women [9], but a higher prevalence has been reported among STD clinic patients i.e. 26% of symptomatic women and 6.5% asymptomatic women tested [9]. In Tanzania and Uganda, a TV prevalence of 41% in Mwanza and 31% in Rakai district, respectively was reported [10]. Most infected persons (70–85%), both men and females have minimal or no symptoms, and untreated infections might last for months to years [11]. Early accurate detection of TV infection in both symptomatic and asymptomatic women plus effective treatment is critical in the prevention of associated complications especially during pregnancy [11].

The diagnosis of TV infection in the current study setting relies on clinical symptoms with or without wet mount microscopy (WMM). Clinical symptoms tend to be non-specific as they cut across a number of other STDs, and are therefore poor indicators of TV infection. The WMM is a rapid and inexpensive test as it involves examination of vaginal fluid in saline under low power microscopy. However, it is associated with limited sensitivity of 38–65% as it depends highly on the expertise of the Microscopist, and prompt processing of samples before the organisms lyse or lose motility, and its sensitivity is even lower among the asymptomatic patients [11–13]. Culture for TV, with sensitivity of 75–96% and a specificity of up to 100%, used to be considered the reference standard for TV diagnosis before molecular tests became available [14]. However, due to its high cost and lengthy time to results of up to 7 days, culture is rarely used in routine setting of resource-poor settings in the diagnosis of TV infection [15].

Recent advancement has led to the development of highly sensitive and specific polymerase chain reaction (PCR)/nucleic acid amplification techniques/tests (NAATs) that offer faster means to detection of TV infection in endocervical, vaginal, or urine specimens from women. These include The APTIMA *T. vaginalis* assay (Hologic Gen-Probe, San Diego, CA), which detects RNA by transcription-mediated amplification with a sensitivity of 95.3–100% and specificity of 95.2–100% [16], the BD Probe Tec TV Qx Amplified DNA Assay (Becton–Dickinson, Franklin Lakes, New Jersey), and the Affirm VP III (Becton–Dickinson, Sparks, MD), a DNA hybridization probe test that evaluates for *T. vaginalis*, *G. vaginalis*, and *Candida albicans,* in only 45 min with sensitivity and specificity of 63 and 99.9%, respectively [17]. Studies on the performance of these PCR assays have mainly been conducted in the developed countries such as USA, Europe, and Australia, and show PCR sensitivity to be 89–98% [18, 19]. These NAATs have become routine assays in developed countries however, in the resource-limited settings the high cost of these commercially available NAATs remains a prohibitive challenge to their use. The objective of this study was to evaluate the technical performance of a cheaper in-house PCR technique in comparison with the conventional WMM against culture as reference standard test for the diagnosis of TV infection in Uganda.

## Methods

This was a cross-sectional prospective and consecutive diagnostic accuracy study conducted from March 2014 to March 2015, at the STD clinic Mulago national referral hospital. Laboratory tests including WMM, PCR and culture were performed at the STD clinic, MBN Clinical Laboratories and Infectious Diseases Institute respectively, all in Kampala, Uganda.

### Participant recruitment

A total of 150 female participants aged 18–60 years attending the STD clinic with symptoms suggestive of TV infection e.g. malodour, abnormal discharge, painful sexual intercourse, dysuria, genital itching and lower abdominal pain voluntarily consented to participate in the study. Exclusion criteria included refusal to consent, being in menstrual period, and history of taking nitro-imidazoles within the past 2 weeks Additional file 1.

### Sample collection

Three vaginal swabs were collected from the posterior fornix by an experienced clinician under a Cusco’s speculum guidance.

### Wet mount microscopy

One vaginal swab was emulsified in three drops of 0.9% saline on a glass slide and examined for motile trichomonads under 100× and 400× microscopy within 30 min of collection as routinely done at the clinic. Microscopic examination was done by one experienced laboratory technician at the STD clinic and by the researcher. Presence of motile trichomonads indicated a positive result for TV infection. No discrepant results were obtained between the two viewers.

### Culture

The second vaginal swab was used within around 20 min to inoculate the InPouch™ TV culture chambers (BioMed Diagnostics, USA) at the STD clinic laboratory following the manufacturer’s instructions. Inoculated chambers were incubated in an upright position at 37 °C for up to 7 days. Microscopic examinations were performed on days 1, 2, 3 5 and 7 to check for the presence of motile trichomonads in the upper chamber (Additional file 2).

### In-house PCR for TV

PCR swabs were temporarily stored up to 3 weeks at minus 20 °C to prepare for batched DNA extraction and PCR assays.

#### DNA extraction

Extraction of TV DNA (if present) was performed using the phenol chloroform method. Briefly, each swab was dipped in sterile 10% sodium dodecyl sulphate (SDS) solution contained in a 1.5 ml eppendorf tube, and heated for 5 min at 65 °C. The swabs were then squeezed against the wall of the tube to express fluids. A volume of 100 µl of sodium acetate was added to neutralize the alkaline component of the lysis solution. A volume of 600 µl of phenol chloroform and 25 µl PCR water was then added. The mixture was vortexed and span at maximum speed (14000 rpm) for 30 min. Purification of extracted DNA was achieved by precipitation using absolute isopropanol. Final DNA precipitate was dissolved in 50 µl of PCR water and stored at −20 °C until amplification.

#### DNA Amplification

Each PCR reaction tube contained 1.0 µl of 25 mmol Magnesium chloride, 1.0 µl, Forward primer 1.0 µl, Reverse primer, 0.1 µl Taq polymerase, master mix with dNTPs 1.0 µl and PCR water 7.0 µl, giving a final volume of 11.1 µl. The forward primer AP65 A_5′GATTCCTCTTCACACACCCACCAG3′ and Reverse primer AP65 B_5′AATACGGCCAGCATCTGTAACGAC3′ were designed to target a conserved region in the AP65 adhesin genes of *T. vaginalis,* giving a 209 base pair (bp) amplification product [20]. Template DNA 2 µl was added to the mixture and loaded onto a Gene Amp PCR System 9700 Thermocycler (Applied Biosystems Inc.). The amplification program included one cycle of initial denaturation at 95 °C for 5 min, followed by 30 cycles of: denaturation at 94 °C for 45 s, annealing at 53 °C for 45 s, extension at 72 °C for 45 s, followed by one cycle of final extension at 72 for 10 min and storage at 4.0 °C.

#### Detection of amplification products

**Fig. 1:**
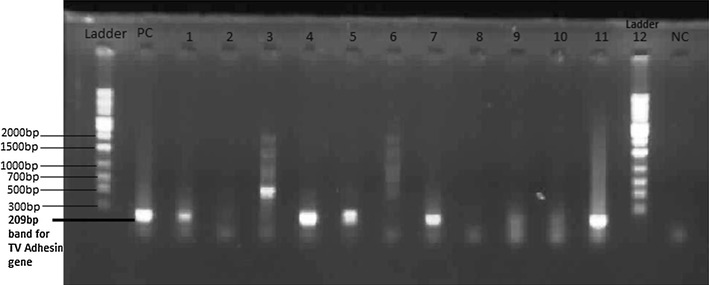
Gel electrophoresis image of post PCR products of the *Trichomonas vaginalis* Adhesin gene. *Lanes* 1 and 12: 1000 bp Ladder, *Lane* 2: Positive control (PC) showing a 209 bp band size, *Lane* 3, 6, 7, 9, and 13: Samples positive for TV, *Lane* 4, 5, 8, 10, 11, and 12: Samples Negative for TV, *Lane* 15: Negative control (NC)

This was performed with 1.5% agarose gel electrophoresis at 120 volts for 45 min. Gel images were viewed under ultraviolet light trans-illuminator and a gel picture taken with a digital UV camera. Presence of 209 bp band on the gel as indicated a positive result for TV (Fig. 1).

#### Quality control

Each batch of PCR included controls at all stages. Positive controls included DNA extracted from a clinical isolate of *Trichomonas vaginalis* grown in vitro, while negative controls consisted of nuclease-free water in the place of template DNA. A 1000 kb ladder (Solis BioDye) was used as a size marker.

## Results

The table showing all the analysed study data is provided as Additional file 3.

### Demographic and clinical characteristics of study participants

The demographics and other clinical details of the studied participants are summarised in Table 1.

**Table 1:** Demographic and clinical characteristics of study participants (N = 150)

Parameter	Categories	Frequency	Percentage
Marital status	Single	81	54.0
	Married	52	34.6
	Separated	9	5.0
	Widowed	4	2.7
	Unknown	4	2.7
Education status	High school	68	55.4
	Unknown	37	24.7
	Tertiary^a^	22	14.7
	Primary level	21	14.0
	None	2	1.3
Abnormal discharge	Yes	125	83.3
	No	25	16.7
Dysuria	Yes	31	20.7
	No	119	79.3
Genital itching	Yes	72	48.0
	No	78	52.0
Lower abdominal pain	Yes	63	42.0
	No	87	58.0
Malodour	Yes	41	27.3
	No	109	72.7
Antibiotic use	Yes	52	34.7
	No	98	65.3

^a^Certificate, diploma and degree awarding institutions

### Prevalence of TV infection

Out of 150 participants, 13 were diagnosed with TV infection using either one, two or all the three tests giving a prevalence of 9% as detailed in Table 2.

**Table 2:** Prevalence of TV infection by each test (*n* = *150*)

Method	Prevalence (%)	(95% CI)
Combined wet mount microscopy, InPouch™ TV culture and in-house PCR	13 (9)	5–14%
Wet mount microscopy (WMM)	3 (2)	1–6%
InPouch™ TV culture	12 (8)	5–13%
In-house PCR	12 (8)	5–13%

It was found that 69% of all the confirmed infection (by culture) cases had an abnormal vaginal discharge, 54% with dysuria, 46% with genital itching, 31% with malodor, and 31% with lower abdominal pain.

### Sensitivity and specificity of WMM and PCR against culture

Using culture as reference standard, the sensitivity, specificity and *kappa* agreement of wet mount microscopy was 25% (95% CI 5.5–57.2), 100% (95% CI, 97.4–100.0), and 0.38 respectively. Corresponding values for the in-house PCR were 91.7% (95% CI, 61.5–99.8), specificity 99.3% (95% CI, 96–100.0) and 0.91 in the detection of TV in high vaginal swabs among symptomatic women in Uganda. See Table 3 for details.

**Table 3:** Accuracy of wet mount microscopy and in-house PCR against Culture for TV diagnosis

Culture									
Test	TP	FP	FN	TN	Sensitivity (95% CI)	Specificity (95% CI)	*Kappa* agreement	PPV (95% CI)	NPV (95% CI)
PCR	11	1	1	137	91.7 (61.5–99.8)	99.3 (96.0–100.0)	0.91	91.6% (60.8–98.7%)	99.3% (95.5–99.9%)
Wet mount	3	0	9	138	25.0 (5.5–57.2)	100 (97.4–100.0)	0.38	100% (CI NA)	93.8% (91.7–95.5%)

*95% CI* 95% Confidence interval, *FN* False negative, *FP* False positive, *NA* Not applicable, *NPV* Negative predictive value, *PPV* Positive predictive value, *TN* True negative, *TP* True positive

## Discussion

The prevalence of TV infection in this study was 8.6% based on all positive tests. Various studies report different prevalence values ranging from 13 to 41% based on the methods used, study settings, social-demographics and symptomatology of studied populations [4, 9, 10]. In high-risk populations such as female sex workers in sub-Saharan Africa (SSA), the prevalence of active TV infection can be as high as 47% [21]. However, the relationship between symptoms and presence of active TV infection also varies since the symptoms are not very specific. In this study, less than 10% of symptomatic women were confirmed with TV infection. Thus, it appears that in the studied population, TV infection diagnosis requires diagnostic algorithms that include highly sensitive laboratory tests. The in-house PCR was highly sensitive and specific for the detection of TV DNA in vaginal swabs, and far out performed the routinely used WMM in the study setting. In our study, the phenol chloroform method was employed for DNA extraction. If costs would allow, it would be better to use extraction columns like silica-membrane-based DNA purification, believed to increase TV detection; consequently the prevalence of trichomoniasis could probably have been higher in our study. Methodological issues can also affect WMM. Studies elsewhere have shown that the low sensitivity of WMM can further be decreased by 20% with delayed microscopic examination for as few as 10 min [22]. In our study, even when the wet preparation was performed at the sample collection site, by the experienced reader who routinely performs the wet preparations at the clinic, its sensitivity (25%) was still poor. Various studies using related but not exactly the same study designs report the sensitivity of WMM to range from around 38 to 65% [4, 11, 18, 23]. Testardini et al. reported sensitivity of WMM of 45.8% in all the patients studied but it increased to 87.5% in symptomatic patients but in their study, one millilitre of physiologic saline was used instead three drops [4]. Use of three drops instead of 1 ml of physiologic saline solution could be a limitation leading to the lower sensitivity of WMM obtained in our study. These results all together indicate the superiority of PCR over WMM.

Two discrepant results emerged between PCR and culture, one of which was culture positive and PCR negative, and the other culture negative and PCR positive. For the PCR negative sample, we suspect that the PCR swab may have been poorly collected, such that extracted DNA was below the limit of PCR detection. Non-specific PCR inhibitors in the sample could also have inhibited amplification. A strategy to find out this would be inclusion of controls for the presence of amplifiable DNA and PCR inhibitors in each clinical sample using PCR for a human gene. If any sample remains negative for this gene, it would not be included in the data analysis. Consequently, the prevalence of trichomoniasis could have been slightly different, and this could be a limitation in our study. Too much DNA could also inhibit amplification requiring dilution of extracted DNA to optimize the PCR reaction. A dilution of the sample could correct the inhibitory effect, so the results could be positive and consequently, the prevalence of trichomoniasis could have been higher. However, when we diluted and repeated the PCR test, the results remained unchanged.

Regarding the PCR-positive culture negative result, this result may not really be a false positive because the PCR has recently been reported to be more sensitive than culture [24]. This result could also be due to inoculation of culture media with already dead organisms in the sample. Since PCR detects presence of DNA, it is not affected by the viability of the microorganisms in the sample. All positive WMM results were also positive with both the PCR and culture results. It appears that to improve the diagnosis of TV infection, combining methods with higher sensitivity such as culture and PCR are recommended, mainly in asymptomatic pregnant women.

### Implications of our findings

Given the high risks of TV infection to pregnancy and increased HIV transmission/acquisition, prompt detection and treatment should be considered critical. Combined methods such as culture and PCR is recommended, since culture depends only on the viability of the microorganism and PCR is unaffected by dead organisms but by inhibitors.

## Conclusion

Among the TV symptomatic women, the sensitivity of the WMM was very low, with two-thirds of the patients missing a diagnosis while the in-house PCR was highly sensitive and specific. Feasibility studies aimed at incorporating PCR tools in algorithms for diagnosis of TV infection among symptomatic women in resource-limited settings are recommended. For WMM, increasing the sample volume and volume of saline to one ml might help to increase the test sensitivity.

## Additional files

**Additional file 1.** InPouch™ TV culture chambers.

**Additional file 2.** Motile trichomonads as seen microscopically in the InPouch™ TV culture chambers (400×).

**Additional file 3.** Dataset analyzed for sensitivity and specificity of WMM and in-house PCR.

## Abbreviations

ELISA: enzyme-linked immunosorbent assay
HIV: human immunodeficiency virus
NAATs: nucleic acid amplification tests
PCR: polymerase chain reaction
Rpm: revolutions per minute
STD: sexually transmitted disease
STI: sexually transmitted infection
TV: *Trichomonas vaginalis*
WHO: World Health Organisation
WMM: wet mount microscopy
µl: micro litre

## References

[1] Van der Pol B (2007). *Trichomonas vaginalis* infection: the most prevalent nonviral sexually transmitted infection receives the least public health attention. Clin Infect Dis.

[2] Cherpes TL, Wiesenfeld HC, Melan MA, Kant JA, Cosentino LA, Meyn LA (2006). The associations between pelvic inflammatory disease, *Trichomonas vaginalis* infection, and positive herpes simplex virus type 2 serology. Sex Transm Dis.

[3] Cotch MF, Pastorek JG, Nugent RP, Hillier SL, Gibbs RS, Martin DH (1997). *Trichomonas vaginalis* associated with low birth weight and preterm delivery. The Vaginal Infections and Prematurity Study Group. Sex Transm Dis.

[4] Testardini P, Vaulet ML, Entrocassi AC, Menghi C, Eliseht MC, Gatta C (2016). Optimization of *Trichomonas vaginalis* diagnosis during pregnancy at a University Hospital, Argentina. Korean J Parasitol..

[5] World Health Organization: Global prevalence and incidence of selected curable sexually transmitted infections. Geneva: WHO Press. 2001.

[6] World Health Organization (1999). The world health report making a difference.

[7] HobbsMM, Sena AC, Swygard H, Schwebke JR. *Trichomonas vaginalis* and trichomoniasis. In: Holmes K, Holmes KK, Sparling PF, Sparling P, Stamm WE, Stamm W, Piot P, Wasserheit, JN, Wasserheit J, Corey L, Cohen MS, Cohen M (Eds). Sexually transmitted diseases. 2008. p. 771–793.

[8] Poole DN, McClelland RS (2013). Global epidemiology of *Trichomonas vaginalis*. Sex Transm Infect..

[9] Sutton M, Sternberg M, Koumans EH, McQuillan G, Berman S, Markowitz L (2007). The prevalence of *Trichomonas vaginalis* infection among reproductive-age women in the United States, 2001–2004. Clin Infect Dis.

[10] Orroth KK, Korenromp EL, White RG, Changalucha J, de Vlas SJ, Gray RH (2003). Comparison of STD prevalences in the Mwanza, Rakai, and Masaka trial populations: the role of selection bias and diagnostic errors. Sex Transm Infect.

[11] Workowski KA, Bolan GA (2015). Sexually transmitted diseases treatment guidelines, 2015. MMWR Recomm Rep..

[12] Radonjic IV, Dzamic AM, Mitrovic SM, Arsic Arsenijevic VS, Popadic DM, Kranjcic Zec IF (2006). Diagnosis of *Trichomonas vaginalis* infection: the sensitivities and specificities of microscopy, culture and PCR assay. Eur J Obstet Gynecol Reprod Biol.

[13] Caliendo AM, Jordan JA, Green AM, Ingersoll J, Diclemente RJ, Wingood GM (2005). Real-time PCR improves detection of *Trichomonas vaginalis* infection compared with culture using self-collected vaginal swabs. Infect Dis Obstet Gynecol..

[14] Nye MB, Schwebke JR, Body BA (2009). Comparison of APTIMA *Trichomonas vaginalis* transcription-mediated amplification to wet mount microscopy, culture, and polymerase chain reaction for diagnosis of trichomoniasis in men and women. Am J Obstet Gynecol..

[15] Fouts AC, Kraus SJ (1980). *Trichomonas vaginalis*: reevaluation of its clinical presentation and laboratory diagnosis. J Infect Dis.

[16] Sosman JM, MacGowan RJ, Margolis AD, Eldridge E, Flanigan T, Vardaman J (2005). Screening for sexually transmitted diseases and hepatitis in 18–29 year-old men recently released from prison: feasibility and acceptability. Int J STD AIDS.

[17] Andrea SB, Chapin KC (2011). Comparison of Aptima *Trichomonas vaginalis* transcription-mediated amplification assay and BD affirm VP III for detection of *T. vaginalis* in symptomatic women: performance parameters and epidemiological implications. J Clin Microbiol.

[18] Simpson P, Higgins G, Qiao M, Waddell R, Kok T (2007). Real-time PCRs for detection of *Trichomonas vaginalis* beta-tubulin and 18S rRNA genes in female genital specimens. J Med Microbiol.

[19] Nathan B, Appiah J, Saunders P, Heron D, Nichols T, Brum R (2015). Microscopy outperformed in a comparison of five methods for detecting *Trichomonas vaginalis* in symptomatic women. Int J STD AIDS.

[20] Alderete JF, O’Brien JL, Arroyo R, Engbring JA, Musatovova O, Lopez O (1995). Cloning and molecular characterization of two genes encoding adhesion proteins involved in *Trichomonas vaginalis* cytoadherence. Mol Microbiol.

[21] Rassjo EB, Kambugu F, Tumwesigye MN, Tenywa T, Darj E (2006). Prevalence of sexually transmitted infections among adolescents in Kampala, Uganda, and theoretical models for improving syndromic management. J Adolesc Health.

[22] Kingston MA, Bansal D, Carlin EM (2003). ‘Shelf life’ of *Trichomonas vaginalis*. Int J STD AIDS.

[23] Wendel KA, Erbelding EJ, Gaydos CA, Rompalo AM (2002). *Trichomonas vaginalis* polymerase chain reaction compared with standard diagnostic and therapeutic protocols for detection and treatment of vaginal trichomoniasis. Clin Infect Dis.

[24] Domeika M, Zhurauskaya L, Savicheva A, Frigo N, Sokolovskiy E, Hallen A (2010). Guidelines for the laboratory diagnosis of trichomoniasis in East European countries. J Eur Acad Dermatol Venereol.

