# Supplementary material for: Very low sensitivity of wet mount microscopy compared to PCR against culture in the diagnosis of vaginal trichomoniasis in Uganda: a cross sectional study
Source: BMC Res Notes. 2017 Jul 6;10:259. doi: 10.1186/s13104-017-2581-1 (PMC5501264; doi:10.1186/s13104-017-2581-1)
Supplement: Supplementary file 1 — Additional file 1. InPouch™ TV culture chambers. [file 13104_2017_2581_MOESM1_ESM.docx]

**Upper**

**Chamber**

**Lower Chamber**

**Additional file 1.**
